# A study on the use of a mechanical shaker for harvesting orange trees

**DOI:** 10.1038/s41598-026-51139-0

**Published:** 2026-05-29

**Authors:** Zeinab Farag Meshref, Mohamed Kadry Abd El Wahab, Ahmed Mohamed El Shal

**Affiliations:** Department of Agricultural Engineering, Zagazig University, Zagazig, 44519 Egypt

**Keywords:** Fabrication, Shaker prototype, Orange, Mechanical, Engineering, Materials science

## Abstract

In this study, a locally fabricated limb-shaking mechanical harvester prototype was developed and field tested to increase the efficiency of orange harvesting and reduce reliance on labor-intensive manual practices. The prototype was evaluated at a fixed vibration amplitude of 50 mm under different tractor PTO speeds (200–450 rpm; 3.33–7.5 Hz) and shaking durations (15–45 s). Fruit detachment efficiency increased significantly with increasing PTO speed and shaking time, whereas shorter shaking durations improved field productivity by allowing more trees to be harvested per hour. Intermediate operating conditions achieved detachment efficiencies comparable to those of the maximum treatment while requiring lower specific energy input. In particular, operation at 350 rpm for 30 s provided an optimal balance between fruit removal efficiency (81.4%), specific energy consumption, and harvesting throughput. These findings demonstrate that moderate PTO speeds and shaking durations can improve the energy efficiency of mechanical orange harvesting while maintaining high detachment performance, supporting more sustainable and cost-effective citrus production. Statistical analysis confirmed that both PTO speed and shaking duration significantly affected fruit detachment efficiency and harvesting productivity.

**Supplementary Information:**

The online version contains supplementary material available at 10.1038/s41598-026-51139-0.

## Introduction

Orange is one of the most important fruit crops in Egypt, where it is considered a vital source of essential vitamins and minerals for the body. Orange is used in the production of juice, oil, and dried fruits as well as in other food industries. Brazil ranks first in orange production worldwide, with a planting area of 575,437 ha, productivity of 30,612.7 kg ha^−1^, and total production of 17,615,667 t. In comparison, Egypt’s orange planting area is 164,010 ha, with a productivity of 22,559.5 kg ha^−1^, and total production is 3,700,000 t^1^. Mechanical citrus harvesting experiments revealed that frequencies between 4.6 and 18.1 Hz and vibration amplitudes from 0.06 to 0.18 m influence fruit detachment, with the removal percentage following a logarithmic trend over time according to field and laboratory studies^2^.

The vibratory behavior of citrus fruits during mechanical harvesting was analysed via slow-motion cameras to better understand fruit detachment mechanisms. Tests on individual fruits with short stem sections using strokes of 60–180 mm and frequencies of 3–18 Hz revealed that some fruits could not be detached at low frequencies combined with short strokes^3^.

Spain is the world’s leading exporter of fresh citrus, where manual harvesting accounts for nearly 50% of total production costs.Mechanical harvesting improves labor productivity and reduces production costs, however, its adoption remains limited^4^.

 Mechanical harvesting could significantly lower production costs, but its implementation by the private sector has been slow. . For frequencies below 10 Hz, three ranges showed maximum vibration transmission: 1.5–2.5 Hz provided the most efficient transmission (> 90% fruit response), 4.5–5 Hz enabled discrimination between mature and immature fruit, and 7–8 Hz represented the lowest third transmission band^5^.

Mature citrus fruits exhibit stronger vibration responses due to their greater mass, with optimal synchronization with shaker rods at 4.1–4.9 Hz and accelerations of 39–60 m s^−2^, resulting in detachment within 1.45–5.75 s. Catch frames and padded components help reduce bruising and canopy damage^6^.

Simulation studies revealed a positive relationship between shaking frequency and stem stress, indicating that a frequency of approximately 5 Hz is sufficient for effective fruit removal^7^.

Field trials conducted at 4.7 Hz achieved 82.6% fruit removal with 5.4% tree damage, whereas combining frequencies of 4.7 Hz and 4.1 Hz further reduced tree damage by 3.9%^8^.

Well-operated mechanical harvesters are capable of removing more than 80% of oranges without causing significant damage to trees^9^.

Dual-sided canopy shakers equipped with catch frames achieved a fruit removal efficiency of 78%, with approximately 70% of the total yield successfully recovered^10^.

Low-frequency shaking (3–6 Hz) with amplitudes of at least 60 mm proved effective with minimal defoliation, although some large commercial machines caused damage to fresh-market fruit. A lightweight tractor-mounted limb shaker achieved 62–97% fruit removal at 3–6 Hz and amplitudes of 100–180 mm^11^.

Computational models of trunk shakers accurately predict vibration modes with less than 4% error under free operation, supporting design optimization and reducing the need for extensive field trials^12^.

Future developments in mechanical harvesting emphasize optimizing vibration parameters, implementing shake-and-catch systems to reduce fruit injury, and integrating postharvest sorting to improve overall efficiency^13^.

A CAD-based multilevel fruit-catching system with four layers reduced the fruit drop distance and minimized fruit damage during pear harvesting, with potential application to soft fruit crops^14^.

Configurable trunk shakers tested on olive, almond, and orange branches at frequencies of 12.25–20.75 Hz and amplitudes of 58–86 mm presented transmissibility losses of 20–60%, with peak amplification reaching 136% in orange branches^15^.

Shock absorbing canvases can effectively reduce puncture and rubbing injuries and has demonstrated potential as a ground-level fruit reception system for fresh-market citrus harvesting^16^. The objectives of this study are to fabricate a shaker prototype for orange harvesting under Egyptian conditions and improve certain operating factors that may affect the efficiency of the fabricated shaker prototype.

The novelty of this work lies in the development and field evaluation of a locally fabricated limb shaker designed specifically for Egyptian citrus orchards, combined with an experimental analysis of the relationships among vibration parameters, fruit detachment efficiency, and energy consumption.

## Materials and methods

The prototype was fabricated in 2022 at a private workshop in El Awamra village, Abu Kebir town, Sharkia Governorate, Egypt. Preliminary testing was conducted during the 2023–2024 season, whereas the main experiments were carried out in Aga, Dakahlia Governorate, Egypt, during the 2024–2025 season. A mechanical limb-shaking system was employed to harvest orange fruits by shaking the tree limbs.

### Materials

#### Machinery and equipment

##### Tractor

A four-wheeled tractor of the standard type was used as a power source with the following specifications:

Type: Kubota engine: three cylinders with four strokes; diesel engine with direct injection; engine power: 18.38 kW (25 hp); Hitch: three points; cooling system: water.

##### Fabricated tree shaker

**Fig. 1:**
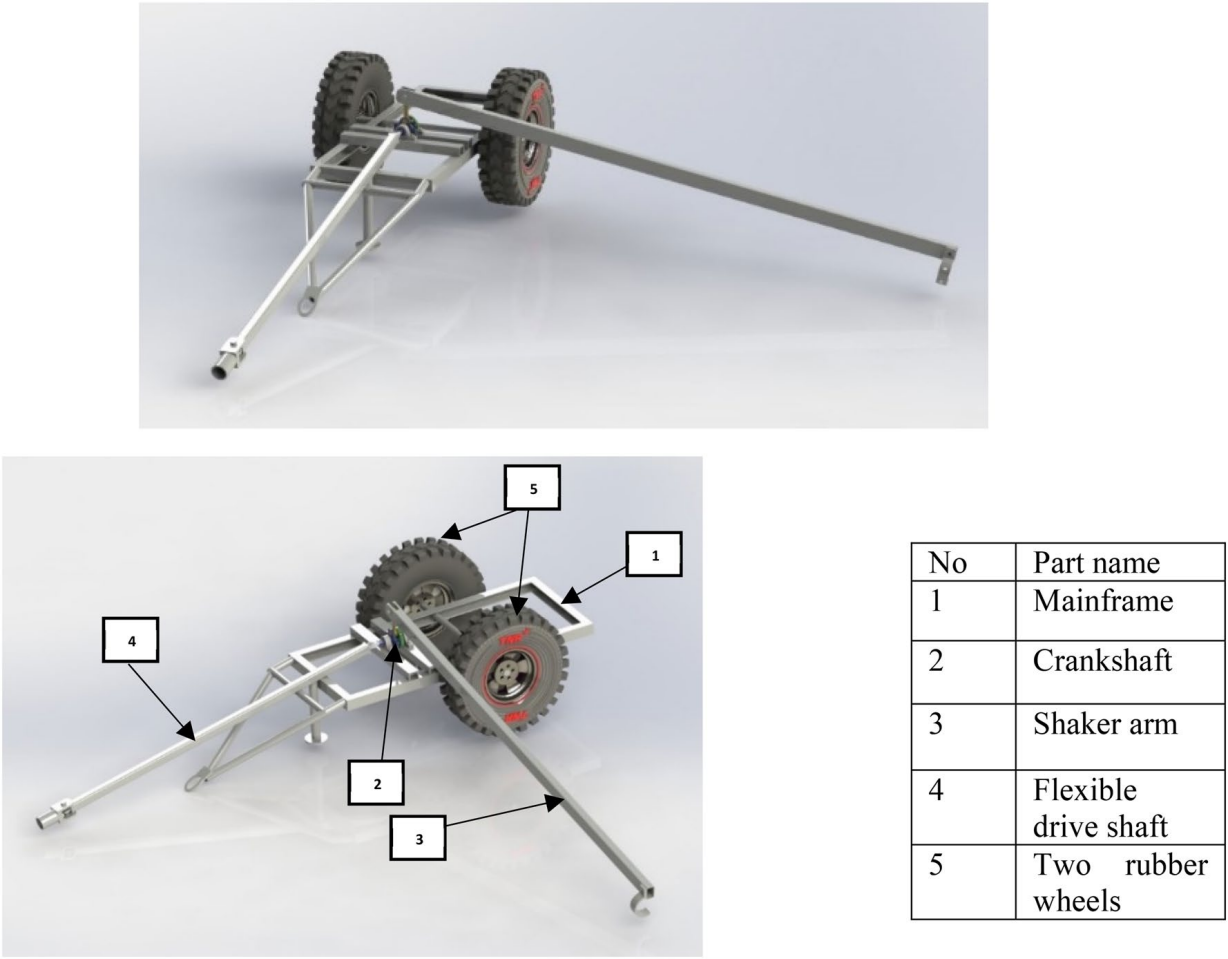
Fabricated shaker prototype isometrics.

**Fig. 2:**
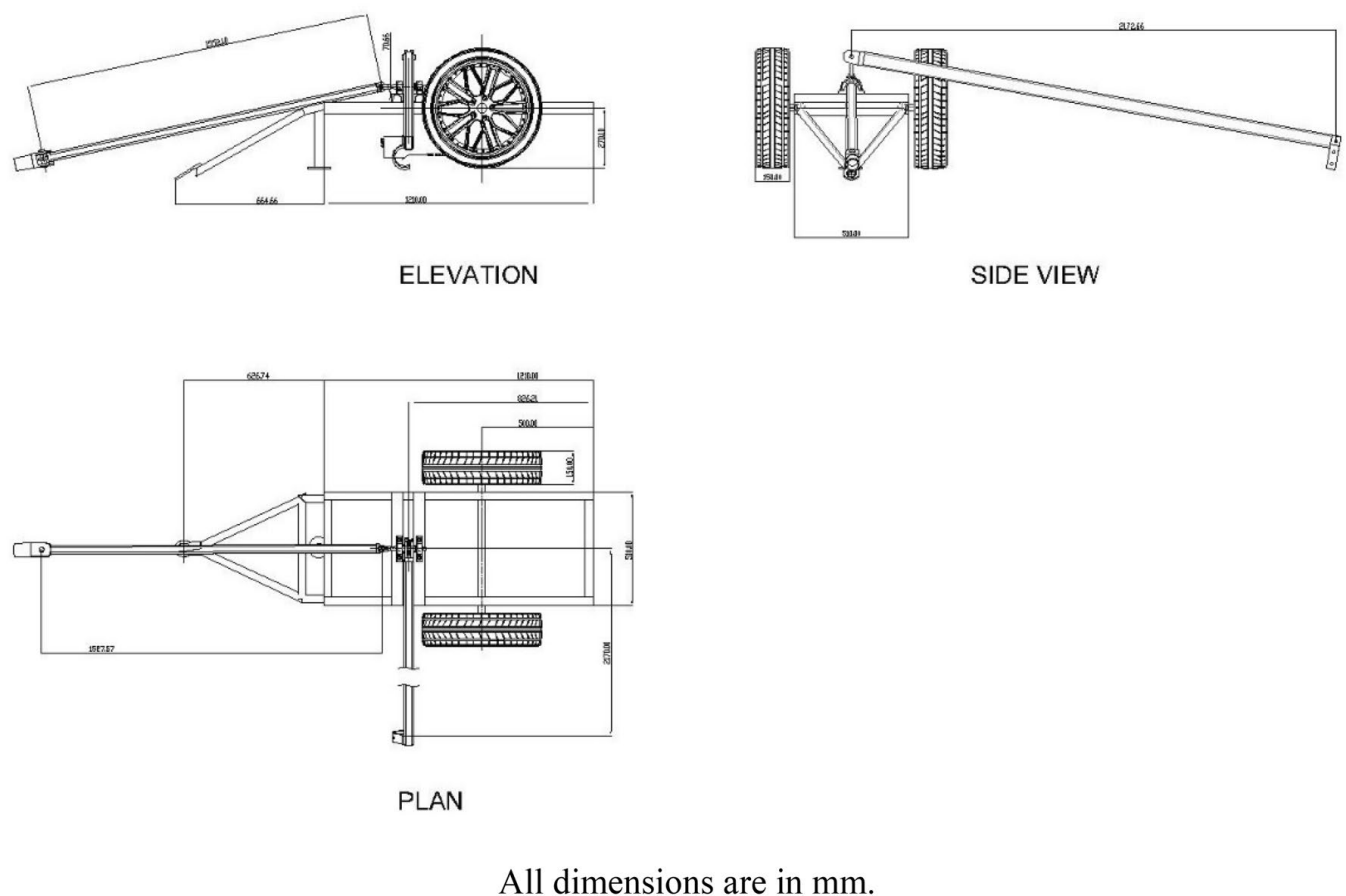
Views of the fabricated shaker prototype**.**

The shaker prototype was manufactured at a private workshop in the village of El Awamra, Abu Kebir town, Sharkia governorate. The fabricated tree shaker prototype consisted of a mainframe, a power transmission system, a crankshaft, and a shaker arm. The fabricated shaker prototype is trailed by a tractor, prototype isometric, elevation, plan, and side view of the prototype, as shown in Figs. 1 and 2. The components of the fabricated prototype shaker are provided in Table 1, the prototype consists of the main structural and functional elements required for operation.

**Table 1:** Components of the fabricated shaker.

No	Part name	About	Pic
1	The main frame	The main frame of the fabricated shaker was constructed from iron bars with dimensions of 1200 mm in length, 513 mm in width, and 360 mm in height on two rubber wheels with dimensions of 550 mm in diameter and 160 mm in width.	
2	Power transmission system	The transmission system of the fabricated shaker transfers the movement of the power take-off connected to a flexible drive shaft with dimensions of 1528 mm length, 50 mm width, and 50 mm height, which is connected to the crankshaft, which is connected by the shaker arm, which vibrates the limbs of the tree.	
3	Crankshaft	The crankshaft was constructed from iron, measuring 220 mm in overall length and featuring a crank radius of 25 mm. It has a single crank throw and is supported by two bearings. These bearings are fixed on the main frame and have dimensions of 60 mm in diameter and 23 mm in width.	
4	Shaker arm	The shaker arm, made of iron and measuring 2170 mm long, 40 mm wide, and 20 mm high, is mounted on the main frame at the vibration point on the tree limb.	

#### Orange trees and orange fruits

The physical and mechanical properties of the Navel orange trees and the orange fruits used in the experiments are provided in Table 2, these properties were determined to support the experimental analysis.

**Table 2:** Physical and mechanical properties of the orange trees and orange fruits.

Characteristic	Average value
Tree height, mm	2500
Trunk diameter, mm	540
Tree age, year	12
Numbers of limbs on tree	4
Distance between rows, mm	5000
Tree distance in the same row, mm	4000
Length of the limb, mm	2100
The length between catching point and the trunk on the limb, mm	800
Limb diameter at vibration catching point, mm	230
Tree yield, kg/year	88
Orange diameter, mm	79.6
Orange length, mm	80
Mass of one orange, g	163.33
Force to remove one ripe orange, N	58.86
The ratio between the removal force and orange mass	36.74

#### Measuring instrument

The measurements and instruments used in the experiment are are provided in Table 3, the table includes images of the devices along with relevant information, descriptions, and their methods of use during the experiment.

**Table 3:** Measuring instrument.

No	Part name	About	Pic
1	Platform scale	An electronic balance for weighing fruits with a maximum balance of 300 kg and an accuracy of 20 g was used.	
2	Tachometer	The revolution per minute of the PTO speed was measured from 0.05–19,999 rpm with ± 0.05% accuracy.	
3	Portable electronic scale	The tensile force in kilograms required to detach fruits from the tree ranged from 10 g to 10 kg with ± 0.05% accuracy.	
4	Fruit-catching canvas	Two thick canopies were placed under the tree; each had dimensions of 4*4 m to prevent the orange from falling on the land.	
5	Graduated cylinder	A graduated cylinder was used to measure fuel consumption for each operation	
6	Measuring tape	A tape measures tree height, trunk diameter, limb diameter, and length and measures distances between trees	
7	Stopwatch	The time consumed for each treatment was measured via a digital stopwatch (Casio JHS) with 1/100-s accuracy to record the time	

### Methods

A practical field experiment was conducted for navel oranges. The evaluation procedures for the fabricated shaker prototype were replicated three times during the harvesting process of nine orange trees. These procedures were conducted at an amplitude of 50 mm, with the following parameters: three PTO speeds (200, 350, and 450 rpm, equivalent to 3.33, 5.83, and 7.5 Hz), and three shaking times (15, 30, and 45 s).

A total of nine treatment combinations were tested (3 PTO speeds × 3 shaking durations). Each treatment was replicated three times to ensure the reliability of the experimental results.

#### Measurements and calculations

##### Detachment efficiency

The detachment efficiency was calculated via the following formula^17^:

1 $${\mathrm{D}}\% = \, \left( {{\mathrm{m}}_{{{\mathrm{hr}}}} /\left( {{\mathrm{m}}_{{\mathrm{r}}} + {\text{ m}}_{{{\mathrm{hr}}}} } \right)} \right) \, \times { 1}00$$

where D is the detached orange percentage, %; m_hr_ is the mass of harvested ripe orange, kg; and m_r_ is the ripe orange mass on the tree that has not fallen, kg.

##### Shaker prototype productivity

Shaker system unit productivity was calculated via the following formula:

2 $${\mathrm{S}}_{{\mathrm{p}}} = {\text{ M}}_{{\mathrm{d}}} /{\mathrm{T}}$$

where S_p_ is the shaker system unit productivity, ton h^−1^; M_d_ is the mass of detached ripe orange, ton; and T is the consumed time, h.

##### Power and specific energy requirements (SER)

Estimating the power as provided by^18^

3 $${\mathrm{P}} = \, \left( {{\mathrm{FC}}/{\mathrm{c}}} \right) \, \times \, \left( {\upeta_{{{\mathrm{th}}}} /{1}00} \right) \, \times {\text{ HV}}$$

where P is the power needed, kW; FC is the consumption fuel, kg h^−1^; η_th_ is the thermal efficiency, %; HV is the heating value of fuel, kJ kg^−1^; and c is equal to 3600.

The specific energy requirements for the shaker prototype are as follows:

4 $${\text{SER }} = {\text{ P}}/{\mathrm{S}}_{{\mathrm{p}}}$$

where SER is the specific energy requirement, kWh ton^−1^; P is the power needed, kW; and S_p_ is the shaker system unit productivity, ton h^−1^.

### Dynamic force analysis of an orange tree shaker with a single crank throw

Mechanical shakers for fruit trees are widely used to facilitate the efficient harvesting of fruit. In this analysis, we consider a tree shaker mechanism that operates via a crankshaft with a single crank throw (eccentric offset). The motion generates reciprocating inertial forces that are transmitted to the tree to induce fruit detachment.

**Fig. 3:**
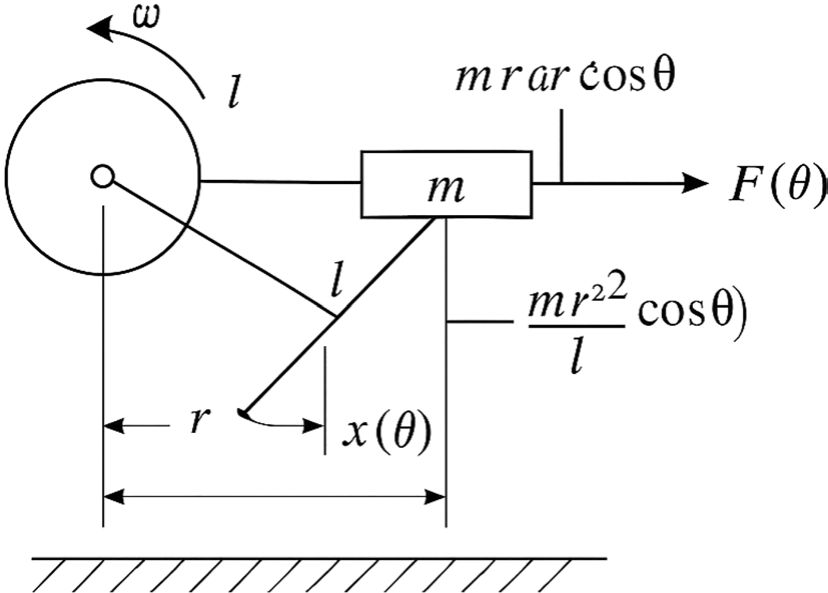
Force diagram of the single crank-throw mechanism^19^.

The system consists of a crankshaft that has a single radius r through a connecting rod that is l in length. Operating at a crankshaft angular velocity ω (rad/s), a reciprocating mass m is connected to the crankshaft by a rod (Fig. 3).

### Kinematic formulas

Assuming pure horizontal motion, the reciprocating mass’s displacement along the horizontal direction is determined by:

5 $$\mathrm{x}(\uptheta ) = \mathrm{r} \mathrm{c}\mathrm{o}\mathrm{s}(\uptheta ) +\sqrt{{l}^{2}-{r}^{2}{\mathrm{s}\mathrm{i}\mathrm{n}}^{2}(\uptheta )}$$

For small crank angles or when l >  > r, the motion can be approximated as:

6 $${\mathrm{x}}\left( \uptheta \right) \, = {\text{ r cos}}\left( \uptheta \right) \, + \, \left( {{\mathrm{r}}^{2}/{\text{2 l}}} \right){\text{ cos}}\left( {{2}\uptheta } \right)$$

### Dynamic force analysis

The inertial force generated by the reciprocating mass is as follows:

7 $${\mathrm{F}}\left( \uptheta \right) \, = \, - {\text{m }}*{{ \ddot {\mathrm{x}}}}\left( \uptheta \right)$$

where the acceleration is approximated by:

8 $${\ddot{\mathrm{x}}}\left( \uptheta \right) \, = \, - {\text{r }}\upomega^{2} {\text{ cos}}\left( \uptheta \right) \, - \, \left( {{\mathrm{r}}^{2} \, \upomega^{2} /{\mathrm{l}}} \right){\text{ cos}}\left( {{2}\uptheta } \right)$$

Thus,

9 $${\mathrm{F}}\left( \uptheta \right) \, = {\text{ m }}\upomega^{2} \, \left[ {{\text{r cos}}\left( \uptheta \right) \, + \, \left( {{\mathrm{r}}^{2}/{\mathrm{l}}} \right){\text{ cos}}\left( {{2}\uptheta } \right)} \right]$$

This includes the primary force m r ω^2^ cos(θ) and the secondary force m (r^2^/l) ω^2^ cos(2θ).

10 $${\text{Dynamic force F}}\left( \uptheta \right) \, = {\text{ m}}\cdot{\mathrm{r}}\cdot\upomega^{{2}} \cdot{\text{ cos}}\left( \uptheta \right)$$

11 $${\text{Secondary force F}}\left( \uptheta \right) \, = {\text{ m}}\cdot \, \left( {{\mathrm{r}}^{{2}} /{\mathrm{l}}} \right) \, \cdot \, \upomega^{{2}} \cdot{\text{ cos}}\left( {{2}\uptheta } \right)$$

## Results and discussion

The results apply specifically to the tested experimental conditions, including the navel orange cultivar, the evaluated PTO speed range (200–450 rpm), the fixed vibration amplitude of 50 mm, and the shaking durations tested in this study. The use of a single cultivar results in a limited vibration amplitude and a restricted frequency range.

### Fruit detachment results

The results indicate that the detachment force varies among different orange varieties, which is consistent with previous studies highlighting the effects of stem thickness and fruit characteristics on detachment ease. A precise methodology was employed to ensure accurate measurements and data analysis, enhancing the reliability of the results and confirming the validity of the field observations.

### Explaining variations between varieties

Some orange varieties require greater detachment forces, likely because of the structural characteristics of the stems and fruits. This variation in results emphasizes that the design of any mechanical harvesting device must consider the physical differences among varieties to ensure optimal efficiency and minimize fruit damage.

### Significance and practical application of the results

This study provides new insights into the efficiency of mechanical harvesting devices. These findings can be applied to improve the design and performance of harvesting equipment, reduce crop losses and increase productivity. Additionally, the results open avenues for future research on the influence of environmental factors and different shaking techniques on fruit detachment rates.

The findings demonstrate that the factors affecting fruit detachment require further investigation to determine the optimal mechanical force and appropriate shaking frequency, which will contribute to better device design and reduced damage. These observations highlight the importance of continued research in this field to ensure practical and effective solutions for farmers.

### Detachment efficiency

**Fig. 4:**
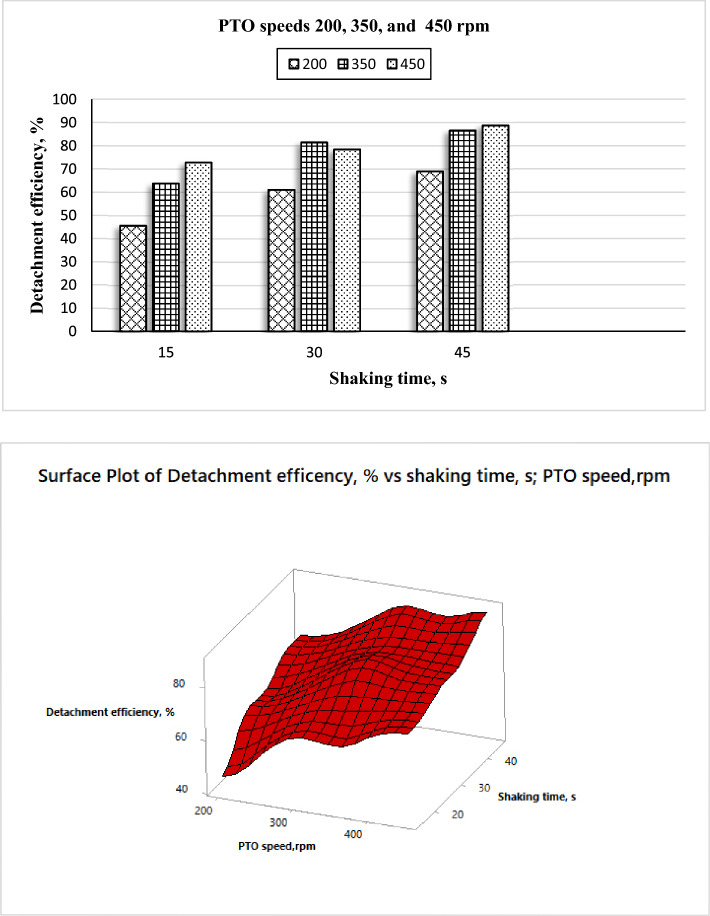
Effects of shaking time on detachment efficiency at PTO speeds of 200, 350, and 450 rpm.

The detachment efficiency increased consistently with both the PTO speed and shaking duration. At the 50 mm amplitude, the efficiency increased from 45.5% at 200 rpm × 15 s to 88.6% at 450 rpm × 45 s, as shown in Fig. 4. Higher speeds and longer shaking times produced greater detachment, although gains beyond 350 rpm or 30 s were modest. No visible damage was observed to fruits, leaves, or branches at any tested setting. These findings are consistent with previous studies on apricots and sweet cherries that reported improved fruit removal with increased excitation time and vibration amplitude.

The increase in detachment efficiency with increasing PTO speed can be explained from a mechanical perspective. According to the dynamic force relationship generated by the crank mechanism, the inertial force transmitted to the tree limb increases with the square of the angular velocity. As the PTO speed increases, the vibration frequency and angular velocity increase accordingly, resulting in greater inertial forces acting on the fruit–stem system. When these forces exceed the stem holding force measured in this study (58.86 N), fruit detachment occurs more easily. Therefore, higher PTO speeds improve the probability of exceeding the critical detachment force required for fruit removal.

Shaking duration also plays an important role in fruit detachment. Increasing the shaking time increases the cumulative vibration energy transmitted to the fruit–stem system. Greater excitation allows the fruit to experience repeated loading cycles, which gradually weaken the attachment between the fruit and the stem until detachment occurs. This explains the gradual increase in detachment efficiency when the shaking duration increased from 15 to 45 s.

These results agree with those of^17^, who reported that the percentage of apricots removed increased as both the frequency and amplitude increased. These results agree with those of^20^, who reported that the efficacy of mechanical shaking in sweet cherry harvesting increased as the cumulative excitation time increased. Variations in trunk diameter, tree size, fruit mass, and maturity may influence the relationship between vibration frequency and the percentage of mature fruit removed^4,21^.

The maximum detachment efficiency obtained in this study (88.6%) is comparable to values reported in previous studies on citrus mechanical harvesting. For example, Sola-Guirado et al.^10^ reported fruit removal efficiencies of approximately 78% when using canopy shaker systems. The slightly higher efficiency observed in the present study may be attributed to the direct transmission of vibration to the limb through the fabricated shaker arm, which improves the effectiveness of energy transfer to the fruit.

The transmission of vibration from the shaker arm to the fruit depends on the dynamic properties of the tree structure. Tree limbs behave as flexible mechanical systems that can amplify or damp vibration depending on their stiffness and mass distribution. Consequently, the effectiveness of fruit detachment is influenced not only by the applied vibration but also by the dynamic response of the tree limb.

### Fabricated shaker prototype productivity

**Fig. 5:**
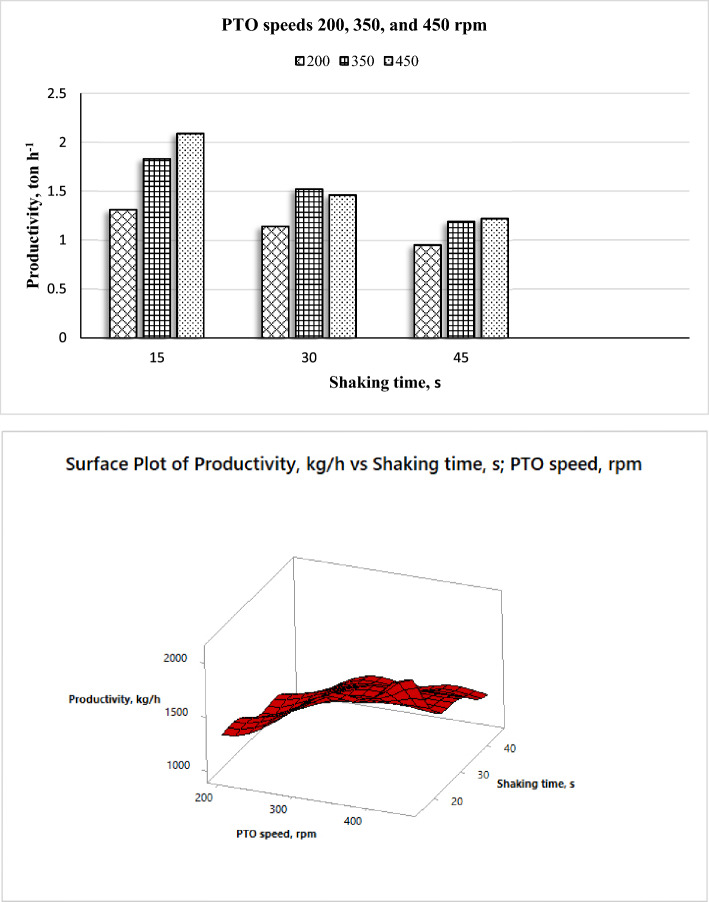
Effect of shaking time on the productivity of the fabricated shaker prototype at PTO speeds of 200, 350, and 450 rpm.

Productivity was inversely related to shaking time but positively correlated with PTO speed, as shown in Fig. 5. At a 50 mm amplitude, increasing the shaking duration from 15 to 45 s reduced the productivity from 1.83 to 1.19 tons h^−1^ at 350 rpm, with similar trends at 200 and 450 rpm. Conversely, increasing the PTO speed from 200 to 450 rpm increased the productivity across all shaking times, peaking at 2.09 tons h^−1^ for the 15 s treatment. The decline in hourly productivity with longer shaking times is attributed to the reduced number of trees processed, outweighing the marginal gain in detachment efficiency per tree.

A trade-off relationship exists between harvesting productivity and detachment efficiency. Although longer shaking durations increase the percentage of detached fruits, they reduce the number of trees harvested per unit time. Therefore, an excessively long shaking duration does not necessarily improve the overall harvesting performance. A moderate shaking duration provides a better balance between fruit removal efficiency and field productivity.

### The power required

**Fig. 6:**
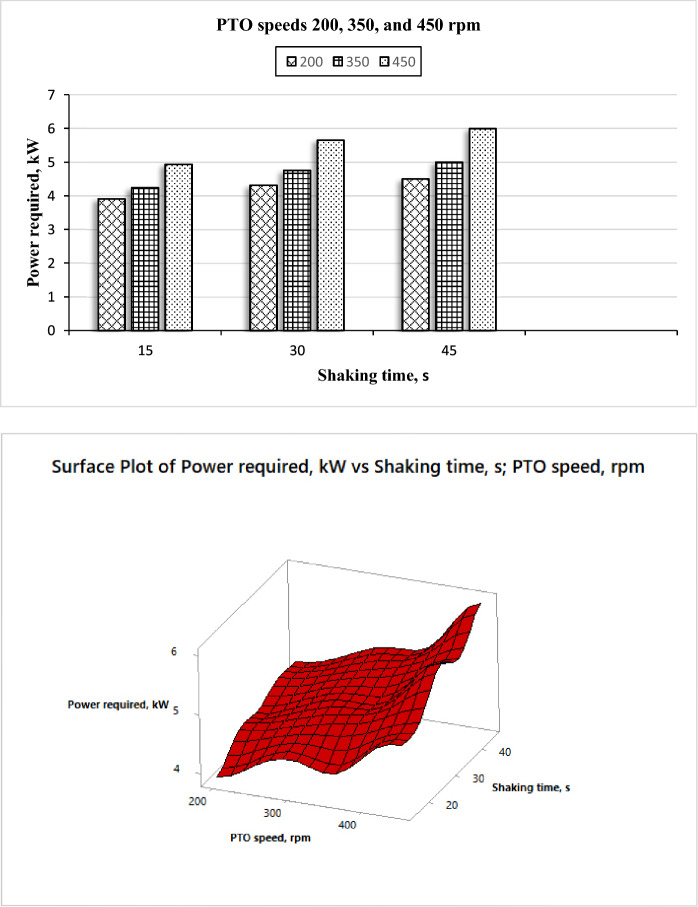
Effect of shaking time on the power required at PTO speeds of 200, 350, and 450 rpm.

At a 50 mm amplitude, extending the shaking time from 15 to 45 s increased the power demand from 3.90 to 4.50 kW, 4.24 to 5.00 kW, and 4.93 to 5.99 kW at PTO speeds of 200, 350, and 450 rpm (3.33, 5.83, and 7.5 Hz), respectively, as shown in Fig. 6. Increasing the PTO speed from 200 to 450 rpm also increased the power consumption at all shaking durations. Longer shaking reduced the number of trees harvested per hour without proportional fruit gain, leading to greater energy use; thus, both a higher PTO speed and prolonged shaking increased the power demand.

The increase in the power requirement with increasing PTO speed is expected because higher rotational speeds generate greater inertial forces and vibration intensities within the shaker mechanism. As the crankshaft rotates faster, more mechanical energy is transferred to the tree limbs, which improves fruit detachment but also increases the mechanical load on the system. This explains the gradual increase in power consumption observed at higher PTO speeds.

### Specific energy requirements

**Fig. 7:**
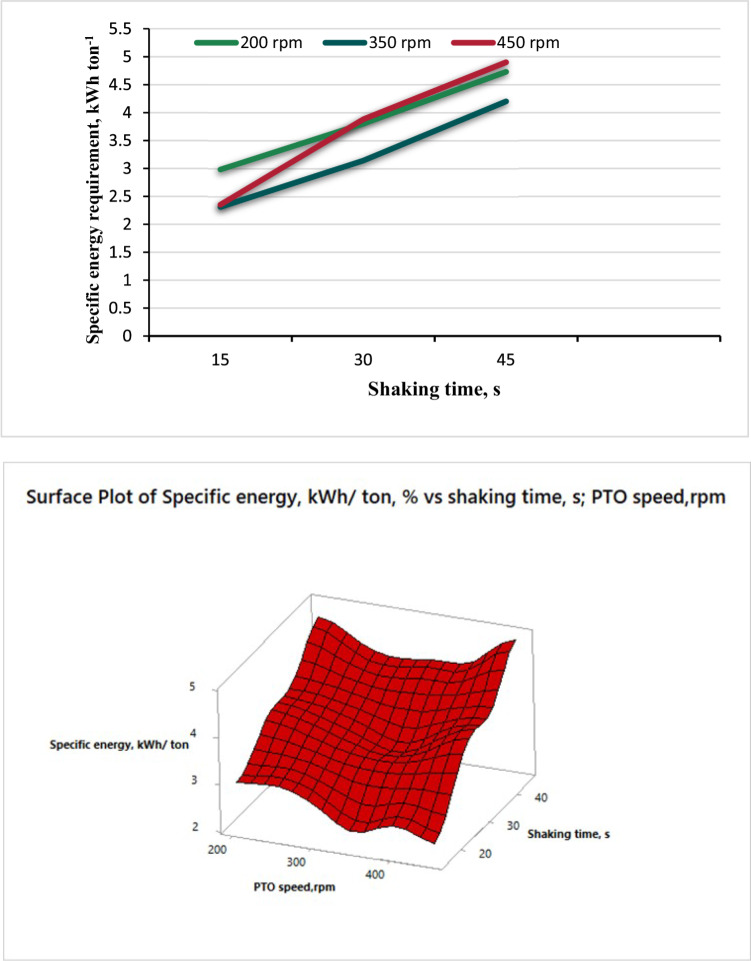
Effects of shaking time on specific energy requirements at PTO speeds of 200, 350, and 450 rpm.

At a 50 mm amplitude, extending the shaking time from 15 to 45 s increased the SER from 2.98 to 4.73, 2.31 to 4.20, and 2.35 to 4.90 kWh ton^−1^ at PTO speeds of 200, 350, and 450 rpm (3.33, 5.83, and 7.5 Hz), respectively, as shown in Fig. 7.

Increasing the PTO speed from 200 to 350 rpm reduced the SER at all shaking durations because the increase in productivity exceeded the increase in power demand. Conversely, increasing the PTO speed from 350 to 450 rpm increased the SER as the extra power consumption exceeded the productivity gain. A longer shaking time also increased the SER by reducing the number of trees harvested per hour.

The 350 rpm × 30 s setting provided a practical balance SER = 3.14 kWh ton^−1^, power = 4.75 kW, detachment = 81.4%, offering near-maximum efficiency with moderate energy use.

From an energy efficiency perspective, operating conditions should not only maximize fruit detachment but also minimize energy consumption per unit of harvested yield. In this study, the operating conditions of 350 rpm and 30 s provided a suitable compromise between detachment efficiency, productivity, and energy consumption. This condition achieved high fruit removal while maintaining moderate specific energy requirements.

### Statistical analysis

Statistical analysis was conducted via Minitab statistical software, and the data were analysed via analysis of variance (ANOVA).

### 2-way ANOVA 95% confidence level

#### General linear model: detach efficiency, % versus PTO… shaking time, s

Method

**Table:** 

Factor coding	(− 1, 0, + 1)

Factor information

**Table:** 

Factor	Type	Levels	Values
PTO speed, rpm	Fixed	3	200, 350, 450
Shaking time, s	Fixed	3	15, 30, 45

Analysis of variance

**Table:** 

Source	DF	Seq SS	Contribution	Adj SS	Adj MS	F Value	P Value
PTO speed, rpm	2	816.55	53.83%	816.55	408.27	37.00	0.003
Shaking time, s	2	656.08	43.26%	656.08	328.04	29.73	0.004
Error	4	44.14	2.91%	44.14	11.03		
Total	8	1516.77	100.00%				

Model summary

**Table:** 

S	R-sq	R-sq(adj)	PRESS	R-sq(pred)
3.32181	97.09%	94.18%	223.448	85.27%

Coefficients

**Table:** 

Term	Coef	SE Coef	95% CI	T Value	P Value	VIF
Constant	71.81	1.11	(68.74, 74.89)	64.85	0.000	
PTO speed, rpm						
200	− 13.38	1.57	(− 17.73, − 9.03)	− 8.54	0.001	1.33
350	5.32	1.57	(0.97, 9.67)	3.40	0.027	1.33
450	8.06	1.57	(3.71, 12.40)	5.14	0.007	*
Shaking time, s						
15	− 11.21	1.57	(− 15.56, − 6.86)	− 7.16	0.002	1.33
30	1.72	1.57	(− 2.63, 6.07)	1.10	0.333	1.33
45	9.49	1.57	(5.14, 13.84)	6.06	0.004	*

Regression equation

$$ \begin{aligned} {\text{Detach efficiency}}, \, \% & = {71}.{81 } - {13}.{38} \;{\mathrm{PTO}} \;{\mathrm{speed}}, {\mathrm{rpm}}\_{2}00 \, + {5}.{32}\;{\text{PTO speed}},\;{\mathrm{rpm}}\_{35}0 \hfill \\ & \quad + {8}.0{6} \;{\mathrm{PTO}} \;{\mathrm{speed}}, {\mathrm{rpm}}\_{45}0 \, - {11}.{21} \;{\mathrm{Shaking}} {\mathrm{time}}, \;{\mathrm{s}}\_{15} \hfill \\ & \quad + { 1}.{\text{72 Shaking time}},{\text{ s}}\_{3}0 \, + {9}.{\text{49 Shaking time}},{\text{ s}}\_{45} \hfill \\ \end{aligned} $$

The results of the two-way ANOVA indicated that both PTO speed and shaking duration had a statistically significant effect on fruit detachment efficiency. The P values for PTO speed (P = 0.003) and shaking duration (P = 0.004) were lower than the significance level (α = 0.05), indicating that changes in these parameters significantly influence fruit detachment performance.

The contribution analysis revealed that the PTO speed accounted for approximately 53.83% of the total variation in the detachment efficiency, whereas the shaking duration contributed approximately 43.26%. These findings indicate that the PTO speed is the most influential factor affecting fruit detachment under the tested conditions.

Furthermore, the high coefficient of determination (R^2^ = 97.09%) indicates that the developed statistical model explains most of the variability in the experimental data, demonstrating a strong relationship between the operating parameters and harvesting performance.


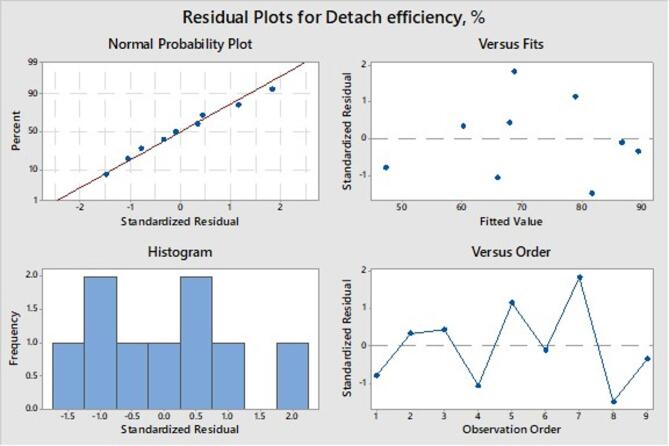


#### General linear model: productivity, kg/h versus PTO… shaking time, s

Method

**Table:** 

Factor coding	(− 1, 0, + 1)

Factor information

**Table:** 

Factor	Type	Levels	Values
PTO speed, rpm	Fixed	3	200, 350, 450
Shaking time, s	Fixed	3	15, 30, 45

Analysis of variance

**Table:** 

Source	DF	Seq SS	Contribution	Adj SS	Adj MS	F Value	P Value
PTO speed, rpm	2	361,716	34.75%	361,716	180,858	8.44	0.037
Shaking time, s	2	593,594	57.02%	593,594	296,797	13.86	0.016
Error	4	85,678	8.23%	85,678	21,420		
Total	8	1,040,988	100.00%				

Model summary

**Table:** 

S	R-sq	R-sq(adj)	PRESS	R-sq(pred)
146.354	91.77%	83.54%	433,746	58.33%

Coefficients

**Table:** 

Term	Coef	SE Coef	95% CI	T Value	P Value	VIF
Constant	1411.9	48.8	(1276.4, 1547.3)	28.94	0.000	
PTO speed, rpm						
200	− 279.9	69.0	(− 471.5, − 88.4)	− 4.06	0.015	1.33
350	101.0	69.0	(− 90.6, 292.5)	1.46	0.217	1.33
450	178.9	69.0	(− 12.6, 370.5)	2.59	0.060	*
Shaking time, s						
15	333.6	69.0	(142.0, 525.2)	4.84	0.008	1.33
30	− 42.4	69.0	(− 234.0, 149.1)	− 0.62	0.572	1.33
45	− 291.2	69.0	(– 482.7, – 99.6)	– 4.22	0.013	*

Regression equation

$$ \begin{aligned} {\mathrm{Productivity}},{\text{ kg}}/{\mathrm{h}} & = {1411}.{9 } - { 279}.{\text{9 PTO speed}},{\text{ rpm}}\_{2}00 \, + { 1}0{1}.0{\text{ PTO speed}},{\text{ rpm}}\_{35}0 \hfill \\ & \quad + { 178}.{\text{9 PTO speed}},{\text{ rpm}}\_{45}0 \, + { 333}.{\text{6 Shaking}} {\mathrm{time}},{\text{ s}}\_{15} \hfill \\ & \quad - { 42}.{\text{4 Shaking time}},{\text{ s}}\_{3}0 \, - { 291}.{\text{2 Shaking time}},{\text{ s}}\_{45} \hfill \\ \end{aligned} $$

With respect to harvesting productivity, both the PTO speed and shaking duration significantly affected system performance. Shaking duration had the greatest contribution to the total variation in productivity (57.02%), indicating that harvesting time is a critical factor controlling field productivity. Shorter shaking durations allow more trees to be harvested per hour, thereby increasing overall system productivity.


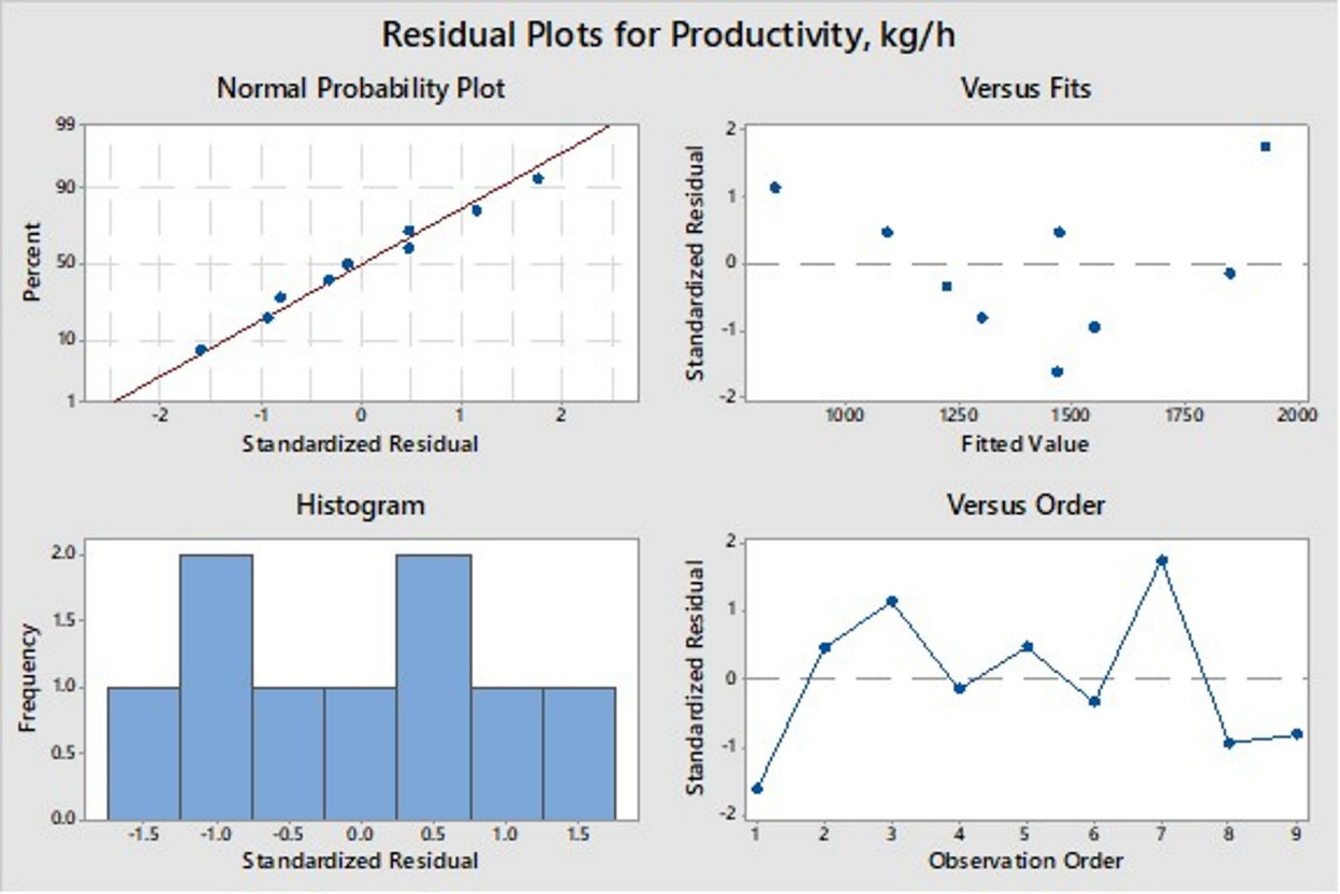


#### General linear model: power needed, kW versus PTO...shaking time, s

Method

**Table:** 

Factor coding	(− 1, 0, + 1)

Factor information

**Table:** 

Factor	Type	Levels	Values
PTO speed, rpm	Fixed	3	200, 350, 450
Shaking time, s	Fixed	3	15, 30, 45

Analysis of variance

**Table:** 

Source	DF	Seq SS	Contribution	Adj SS	Adj MS	F Value	P Value
PTO speed, rpm	2	2.57716	70.58%	2.57716	1.28858	90.67	0.000
Shaking time, s	2	1.01716	27.86%	1.01716	0.50858	35.79	0.003
Error	4	0.05684	1.56%	0.05684	0.01421		
Total	8	3.65116	100.00%				

Model summary

**Table:** 

S	R-sq	R-sq(adj)	PRESS	R-sq(pred)
0.119210	98.44%	96.89%	0.287775	92.12%

Coefficients

**Table:** 

Term	Coef	SE Coef	95% CI	T Value	P Value	VIF
Constant	4.8078	0.0397	(4.6975, 4.9181)	120.99	0.000	
PTO speed, rpm						
200	− 0.5711	0.0562	(− 0.7271, − 0.4151)	− 10.16	0.001	1.33
350	− 0.1444	0.0562	(− 0.3005, 0.0116)	− 2.57	0.062	1.33
450	0.7156	0.0562	(0.5595, 0.8716)	12.73	0.000	*
Shaking time, s						
15	− 0.4511	0.0562	(− 0.6071, − 0.2951)	− 8.03	0.001	1.33
30	0.0956	0.0562	(− 0.0605, 0.2516)	1.70	0.164	1.33
45	0.3556	0.0562	(0.1995, 0.5116)	6.33	0.003	*


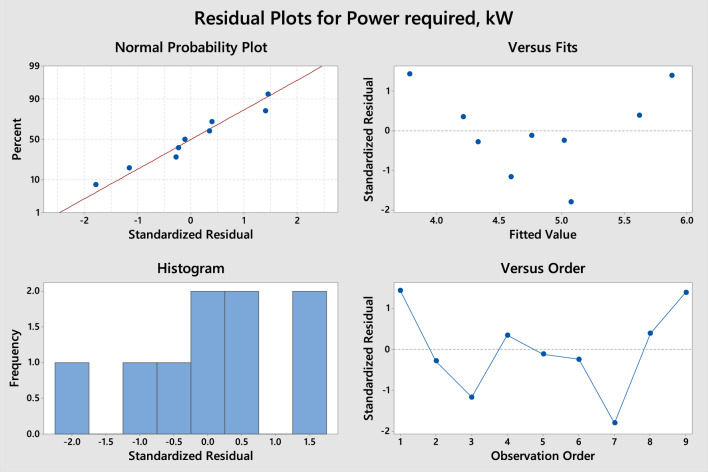


### ANOVA for power requirement

The results of the two-way ANOVA indicated that both PTO speed and shaking duration had a statistically significant effect on the power requirement (P < 0.01). PTO speed is the most effective and significant factor affecting the power requirement, contributing to 70.58%, followed by shaking duration with a contribution of 27.86%. These findings indicate that increasing PTO speed leads to a noticeable increase in the required power during the harvesting process. From the normal probability plot, it is clear that the residuals lie close to the straight line, confirming the adequacy of the statistical model and the significance of the studied variables. Furthermore, the high coefficient of determination (R² = 98.44%) indicates that the developed statistical model explains most of the variability in the experimental data, demonstrating a strong relationship between the operating parameters and the power requirement.

#### General linear model: specific energy, kW.h.ton^−1^, versus shaking time, s

Method

**Table:** 

Factor coding	(− 1, 0, + 1)

Factor information

**Table:** 

Factor	Type	Levels	Values
PTO speed, rpm	Fixed	3	200, 350, 450
Shaking time, s	Fixed	3	15, 30, 45

Analysis of variance

**Table:** 

Source	DF	Seq SS	Contribution	Adj SS	Adj MS	F Value	P Value
PTO speed, rpm	2	0.6438	8.86%	0.6438	0.32191	5.48	0.072
Shaking time, s	2	6.3876	87.90%	6.3876	3.19381	54.34	0.001
Error	4	0.2351	3.24%	0.2351	0.05878		
Total	8	7.2666	100.00%				

Model summary

**Table:** 

S	R-sq	R-sq(adj)	PRESS	R-sq(pred)
0.242441	96.76%	93.53%	1.19025	83.62%

Coefficients

**Table:** 

Term	Coef	SE Coef	95% CI	T Value	P Value	VIF
Constant	3.5878	0.0808	(3.3634, 3.8122)	44.40	0.000	
PTO speed, rpm						
200	0.249	0.114	(− 0.068, 0.566)	2.18	0.095	1.33
350	− 0.371	0.114	(− 0.688, − 0.054)	− 3.25	0.031	1.33
450	0.122	0.114	(− 0.195, − 0.440)	1.07	0.345	*
Shaking time, s						
15	− 1.041	0.114	(− 1.358, − 0.724)	− 9.11	0.001	1.33
30	0.019	0.114	(− 0.298, 0.336)	0.17	0.877	1.33
45	1.022	0.114	(0.705, 1.340)	8.94	0.001	*

Regression equation

$$$$$$ \begin{aligned} {\text{Specific energy}},{\text{ kW.h.ton}^{-1}} & = {3.5878} + {0.249}\:{\text{PTO speed}},{\text{ rpm}}\_{2}00 \, - { 0.371}\:{\text{ PTO speed}},{\text{ rpm}}\_{35}0 \hfill \\ & \quad + { 0122}{\text{ PTO speed}},{\text{ rpm}}\_{45}0 \, - { 1.041}{\text{ Shaking}}\: {\mathrm{time}},{\text{ s}}\_{15} \hfill \\ & \quad + { 0.019}{\text{ Shaking time}},{\text{ s}}\_{3}0 \, + { 1.022}{\text{ Shaking time}},{\text{ s}}\_{45} \hfill \\ \end{aligned} $$$$$$

Statistical analysis of specific energy requirements revealed that shaking duration had a highly significant effect on specific energy requirement (P = 0.001), whereas the effect of PTO speed was less pronounced. This result indicates that longer vibration durations increase , mainly because of the reduction in harvesting productivity and increased power consumption.


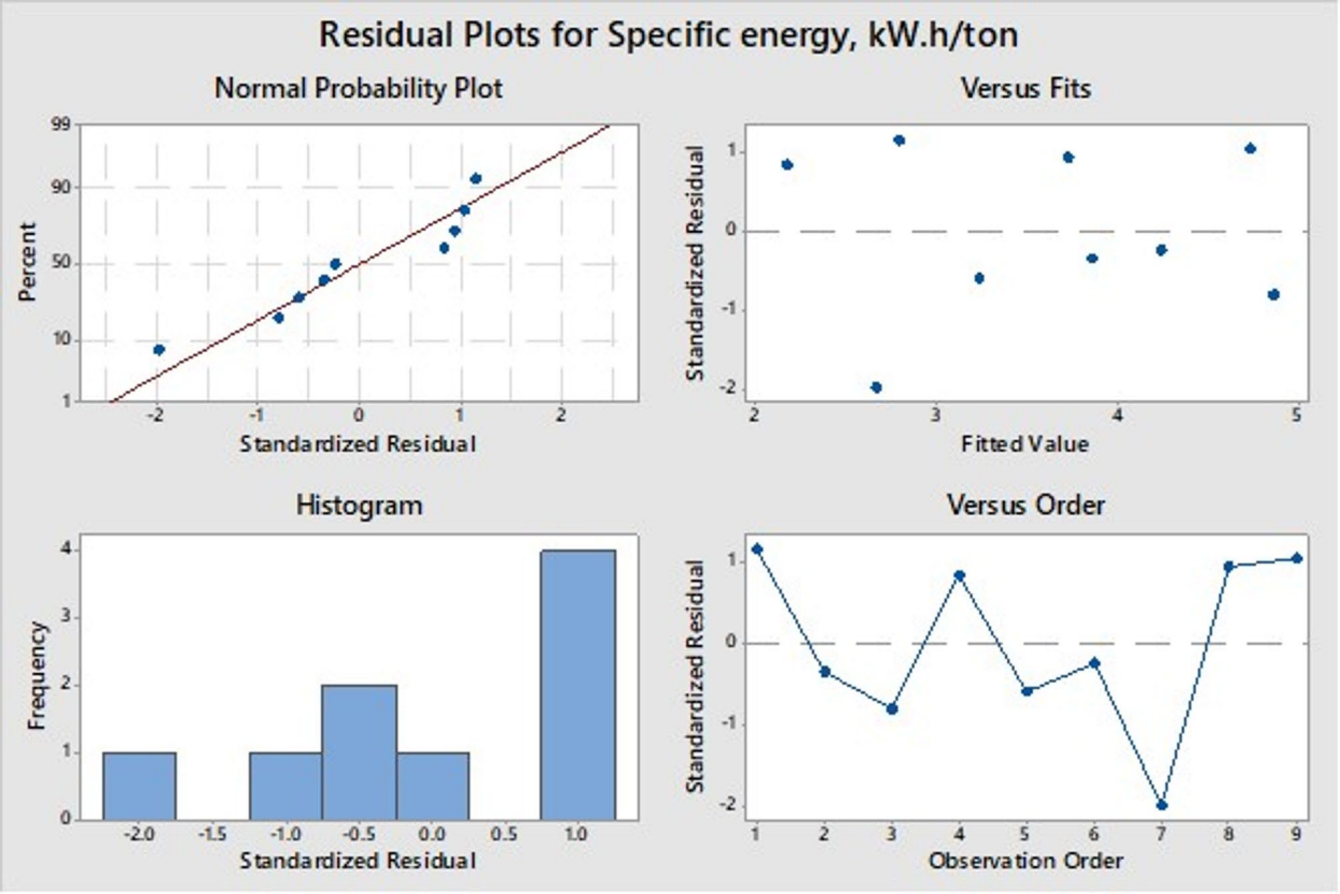


Analysis of variance (ANOVA) was conducted to evaluate the effects of the operating parameters, including the PTO speed and shaking time, on the fruit detachment efficiency, field productivity, and specific energy consumption. The statistical analysis revealed that the studied factors significantly influenced the performance of the developed mechanical harvester.

The results indicated that increasing the PTO speed generally improved the fruit detachment efficiency because of the higher vibration frequency transmitted to the tree branches.

Similarly, the shaking time had a noticeable effect on the detachment process, as longer vibration durations allowed more fruits to overcome the stem holding force and detach from the branches.

The statistical model also revealed that the combined effect of PTO speed and shaking time plays an important role in determining the overall harvesting performance.

Among the tested treatments, the operating conditions of 350 rpm and 30 s provided a balanced performance, achieving high fruit detachment efficiency with moderate energy consumption and acceptable field productivity.

These results confirm that selecting appropriate operating parameters is essential for optimizing the performance and energy efficiency of a mechanical orange harvesting system.

The statistical analysis also revealed that both PTO speed and shaking duration significantly influence fruit detachment efficiency and harvesting productivity. The interaction between these factors suggests that the effect of the PTO speed becomes more pronounced at longer shaking durations. This interaction highlights the importance of selecting appropriate combinations of vibration parameters rather than optimizing each factor independently.

The high values of R^2^ and adjusted R^2^ indicate that the developed statistical models are reliable for describing the relationship between operating parameters and harvesting performance. These results confirm that the selected experimental factors adequately represent the main variables affecting the performance of the fabricated shaker prototype.

The developed regression equations can be used to predict harvesting performance within the tested range of operating parameters. Such predictive relationships are useful for selecting suitable operating conditions in practical field applications without conducting additional experiments.

### Manual and mechanical harvesting of citrus

A calculated operating cost of 3.58 $ h^−1^ and a production cost of 2.36 $ ton^−1^ were achieved under the prototype’s predetermined optimal conditions (350 rpm, 30 s), underscoring the direct economic benefit of operating the harvester at its peak technical performance.

To achieve fruit detachment from the stem, the tensile force must exceed the stem’s holding force. This was ensured by the shaker used. Field experiments were conducted to measure the stem holding force for different orange types. The average forces were calculated and applied to achieve successful detachment by generating a tensile force greater than the holding force.

Regarding energy transfer efficiency from the shaker to the fruits, initially, the shaker receives motion from the tractor’s power take-off (PTO). The PTO drives the crankshaft via flexible coupling. The motion is then transferred to the shaking arm attached to the crankshaft and to the main branch of the tree, converting it into vibrations that act on the fruits. The energy delivered to the fruits can be controlled via PTO. Multiple field trials at different speeds and durations were conducted to optimize energy transfer, efficiency, and force, ensuring fruit detachment without causing damage to the crop or trees.

A direct relationship exists between mechanical vibration and fruit detachment. Field trials were conducted at three PTO speeds: 200, 350, and 450 rpm, and three vibration durations: 15, 30, and 45 s. The results revealed that increasing the vibration intensity and duration increased the number of detached fruits, confirming the positive correlation between vibration and fruit detachment.

This study aimed to conduct practical and scientific trials on the harvesting of different orange varieties at various times and under various conditions, with a focus on “navel” oranges during the midharvest season. The trials ensured harvesting without crop damage and aimed to save time, labor, and costs compared with traditional manual methods. This research also provides technological insights for agricultural engineering to optimize harvesters for field conditions.

Replicates and experimental factors: Nine trees were used with three repetitions. The experimental factors included three PTO speeds and three vibration durations, which were tested and analysed as described.

Measurement methods: Fuel consumption was measured by filling the tractor tank to full capacity and recording consumption via a graduated cylinder after each step. Measurements were taken during operation at different speeds and durations and while idle or moving.

Harvesting time: measured via a stopwatch. Specific durations of 15, 30, and 45 s were set for vibration, and other travel times were recorded practically.

Calibration of instruments: All devices were calibrated in the field, including a tachometer for PTO rotations (200, 350, and 450 rpm) and devices for measuring fruit tensile force.

Compared with conventional manual harvesting, the use of a mechanical shaker significantly reduces labor requirements and harvesting time. Manual harvesting of citrus typically requires a large seasonal workforce and represents a considerable portion of total production costs. Therefore, the developed prototype can contribute to improving economic efficiency in citrus orchards, particularly in regions facing labor shortages.

The results of this study demonstrate the potential of locally fabricated mechanical harvesting systems for citrus production under Egyptian conditions. The developed prototype provides a practical and cost-effective alternative to imported harvesting machinery. Moreover, understanding the relationship between vibration parameters and fruit detachment behavior contributes to improving the design and optimization of future mechanical harvesters.

Despite the promising results obtained in this study, several limitations should be considered. The experiments were conducted on a limited number of trees and focused on a single citrus cultivar under specific field conditions. Additionally, environmental factors such as canopy density, fruit maturity stage, and branch stiffness may influence vibration transmission and fruit detachment efficiency. Therefore, further studies are needed to evaluate the performance of the developed shaker under different orchard conditions and with different citrus varieties.

## Conclusion

This study aimed to design, fabricate, and evaluate a prototype shaker for orange harvesting. Field and scientific experiments were conducted on different orange varieties under various operating conditions and harvesting periods to determine the most suitable operating parameters that achieve high harvesting efficiency with minimal losses in power and specific energy while avoiding fruit damage. The results indicated that Navel orange trees harvested during midseason in January represent the most favourable period for mechanical harvesting in Egypt because of the naturally low fruit detachment force during this period. The results under a fixed vibration amplitude (50 mm), restricted frequency range (3.33–7.5 Hz), and lack of quantitative fruit damage assessment. The results revealed that harvesting efficiency increased with increasing PTO speed and shaking duration, whereas productivity decreased when the shaking time exceeded 30 s due to the reduced number of trees harvested per unit time. The optimal operating conditions were identified as a PTO speed of 350 rpm, a shaking duration of 30 s, and a vibration amplitude of 50 mm, achieving a fruit detachment rate of 81.4%, with moderate specific energy requirements of 3.14 kWh t^−1^ and an acceptable productivity of 1.52 t h^−1^. The experiments were carried out on nine trees arranged in three replications, with three main operating factors, namely, PTO speed, shaking duration, and vibration amplitude, to evaluate their effects on fruit detachment efficiency, specific energy requirements, and productivity. Compared with conventional manual harvesting, mechanical harvesting contributes to reducing the harvesting time, labor requirements, and operational costs while minimizing the drawbacks associated with human labor. This work highlights the importance of applying agricultural engineering principles in the design of harvesting machinery adapted to local field conditions. The prototype successfully achieved scientifically measurable objectives through experimental evaluation, in addition to technological objectives related to the design and development of mechanical harvesting systems, reflecting the growing trend toward agricultural mechanization as a practical and forward-looking approach. A calculated operating cost of 3.58 $ h^−1^ and a production cost of 2.36 $ ton^−1^ were achieved under the prototype’s predetermined optimal conditions (350 rpm, 30 s), underscoring the direct economic benefit of operating the harvester at its peak technical performance. The machine’s performance is valid only under the tested conditions: mid-January in the Nile Delta, for navel orange varieties, with trees of medium to small size and average height. Field experiments were conducted to obtain these results. All the reported figures were obtained under controlled conditions, and repeated trials were performed to ensure accuracy. Further research, including testing the prototype in different orchards and seasons, evaluating performance under larger scale field conditions and assessing fruit and tree damage under extended operation, is recommended.

The results suggest that the fabricated shaker prototype can be considered a promising solution for improving citrus harvesting efficiency under Egyptian field conditions, with potential for further development and large-scale adoption.

Future work should focus on optimizing the vibration amplitude and evaluating fruit damage and machine performance under large-scale commercial harvesting conditions.

## Supplementary Information

Supplementary Information.

## Data Availability

The datasets used and/or analysed during the current study are available from the corresponding author upon reasonable request.
